# Real opinions on general medicine residency programs in Japan: Perspectives from medical students, residents, and young academic generalists

**DOI:** 10.1002/jgf2.584

**Published:** 2022-09-30

**Authors:** Kosuke Ishizuka, Hiroyuki Nagano, Taiju Miyagami, Tatsushi Toyooka, Sunsuke Ohara, Erica Ogami

**Affiliations:** ^1^ Department of General Medicine Chiba University Hospital Chiba Japan; ^2^ Department of Healthcare Economics and Quality Management, Graduate School of Medicine Kyoto University Kyoto Japan; ^3^ Department of General Medicine, Faculty of Medicine Juntendo University Tokyo Japan; ^4^ Community Based Medicine Fujita Health University Aichi Japan; ^5^ School of Medicine Saitama Medical University Saitama Japan

**Keywords:** family doctor, general medicine, hospital general medicine, residency program

## Abstract

Medical students and junior residents have five concerns about general medicine training, and senior residents and young academic generalists respond to these concerns. We hope that this paper will help to dispel some common concerns for those who wish to become specialists in general medicine.

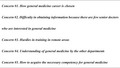

To the Editor,

In Japan, general medicine was added as the nineteenth basic area of medical specialty, and training for specialists in general medicine began in April 2018.^1^ The training consists of General Medicine Specialist Training I (in clinics and small‐ and medium‐sized hospitals), General Medicine Specialist Training II (in general medicine departments of hospitals), Internal Medicine Training, Pediatric Training, and Emergency Medicine Training.^1^ Furthermore, remote training is a requirement of the program.^1^ We conducted interactive interviews and a narrative review of literatures. Consequently, medical students and junior residents have five concerns about general medicine training, and senior residents and young academic generalists answered (Table 1).

**TABLE 1:** Five concerns on medical students and junior residents about training in general medicine

**Concern #1. How general medicine career is chosen**
**Concern #2. Difficulty in obtaining information because there are few senior doctors who are interested in general medicine**
**Concern #3. Hurdles in training in remote areas**
**Concern #4. Understanding of general medicine by the other departments**
**Concern #5. How to acquire the necessary competency for general medicine**

**Concern #1. How general medicine career is chosen**

**Answer:**

General medicine career aspirations are reported to be associated with clinical diagnostic reasoning, community‐oriented practice, and preventive medicine.^2^ The appeal of general medicine training lies in the fact that it encompasses communication skills, consideration of the patient's family and social background, and the “non‐medical” part of medical care as a specialized field of study.^3^

**Concern #2. Difficulty in obtaining information because there are few senior doctors who are interested in general medicine**

**Answer:**

Even those who are interested in general medicine training often express their concerns about the lack of information for future career paths.^1^ It is important to create a platform where medical students and general medicine doctors can be closely involved. The system of general medicine training is being improved daily, and opinions from general medicine residents are being actively collected, gradually dispelling this concern. If the number of residents who have completed general medicine training increases, the concerns may be alleviated.

**Concern #3. Hurdles in training in remote areas**

**Answer:**

In addition to real‐time remote communication using teleconference, remote instruction that overcomes time differences by utilizing SNS such as Slack is useful because the time for meeting is often difficult for both mentors and mentees in clinical settings.

**Concern #4. Understanding of general medicine by the other departments**

**Answer:**

It is very important to convey the joy of clinical practice and to young doctors and medical students. Also, in order to be recognized by other departments in the future, it is important to accumulate research achievements,^4^ making the strengths of general medicine training easier to understand for potential trainees as well as specialists in other fields.

**Concern #5. How to acquire the necessary competency for general medicine**

**Answer:**

Portfolios and experience records from training are important because they allow one to reflect on one's own failures and growth.^1^

It is important to select a training facility that meets the senior residents' needs in terms of mentoring and guidance system and career development.^5^ Furthermore, it is important to promote more collaborative movements between facilities to encourage the young doctors and medical students to receive training at different facilities.

We hope that this paper will help to dispel concerns for those who wish to become specialists in general medicine.

## AUTHOR CONTRIBUTIONS

All authors had access to the data and a role in writing the manuscript.

## CONFLICT OF INTEREST

The authors have stated explicitly that there are no conflicts of interest in connection with this article.
